# Missing value imputation for epistatic MAPs

**DOI:** 10.1186/1471-2105-11-197

**Published:** 2010-04-20

**Authors:** Colm Ryan, Derek Greene, Gerard Cagney, Pádraig Cunningham

**Affiliations:** 1 School of Computer Science and Informatics, University College Dublin, Dublin, Ireland; 2 Conway Institute of Biomolecular and Biomedical Research, University College Dublin, Dublin, Ireland

## Abstract

**Background:**

Epistatic miniarray profiling (E-MAPs) is a high-throughput approach capable of quantifying aggravating or alleviating genetic interactions between gene pairs. The datasets resulting from E-MAP experiments typically take the form of a symmetric pairwise matrix of interaction scores. These datasets have a significant number of missing values - up to 35% - that can reduce the effectiveness of some data analysis techniques and prevent the use of others. An effective method for imputing interactions would therefore increase the types of possible analysis, as well as increase the potential to identify novel functional interactions between gene pairs. Several methods have been developed to handle missing values in microarray data, but it is unclear how applicable these methods are to E-MAP data because of their pairwise nature and the significantly larger number of missing values. Here we evaluate four alternative imputation strategies, three local (Nearest neighbor-based) and one global (PCA-based), that have been modified to work with symmetric pairwise data.

**Results:**

We identify different categories for the missing data based on their underlying cause, and show that values from the largest category can be imputed effectively. We compare local and global imputation approaches across a variety of distinct E-MAP datasets, showing that both are competitive and preferable to filling in with zeros. In addition we show that these methods are effective in an E-MAP from a different species, suggesting that pairwise imputation techniques will be increasingly useful as analogous epistasis mapping techniques are developed in different species. We show that strongly alleviating interactions are significantly more difficult to predict than strongly aggravating interactions. Finally we show that imputed interactions, generated using nearest neighbor methods, are enriched for annotations in the same manner as measured interactions. Therefore our method potentially expands the number of mapped epistatic interactions. In addition we make implementations of our algorithms available for use by other researchers.

**Conclusions:**

We address the problem of missing value imputation for E-MAPs, and suggest the use of symmetric nearest neighbor based approaches as they offer consistently accurate imputations across multiple datasets in a tractable manner.

## Background

Epistatic miniarray profiles (E-MAPs) provide a high-throughput methodology to quantitatively measure the strength of pairwise genetic interactions. Given a pre-defined set of genes, the procedure supports the identification of both positive (alleviating) and negative (aggravating) interactions between genes, assignments that are immensely valuable in interpreting the biological basis of the epistatic relationships [1]. Most commonly an E-MAP is represented in the form of a symmetric matrix, with real-valued entries indicating the type and strength of interaction between each pair of genes under consideration. These scores are calculated based on the divergence in growth of yeast strains with two disrupted genes from the expected growth rate. Typically a normalization process is applied to the interaction scores so that positive matrix entries denote an alleviating interaction, negative matrix entries denote an aggravating interaction, and values close to zero indicate the probable absence of an interaction between two genes - *i.e*. they function in independent pathways in the cell. Full details of the experimental procedure and the normalization process are described in Collins *et al *[2].

**Figure 1:**
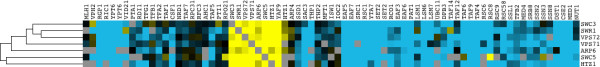
**Swr1 cluster - genes displaying similar interaction profiles**. An example of coherence taken from the Chromosome Biology E-MAP. Members of the Swr1 complex display similar interaction profiles, and as a result are clustered together.

Computational techniques such as cluster analysis may subsequently be applied to the E-MAP score matrix. This type of analysis often provides insight into the underlying biology. For example, subsets of genes with similar interaction profiles may signify complexes of proteins involved in common biological processes [3]. An example of this is shown in Figure 1, where members of the Swr1 complex and the histone HTZ1 all display similar interactions with a variety of genes.

Recently additional techniques for the analysis of E-MAPs have been developed. Pu *et al *[4] have extended the concept of profile similarity using a biclustering approach - so that clusters of genes can be identified which do not necessarily share globally similar interaction profiles, but have a strong coherence over a fraction of their interactions. Ulitsky *et al *[5] and Bandyopadhyay *et al *[1] have developed methods which combine physical interaction data with genetic interaction data in order to identify functional modules and the connections between them.

One common characteristic of E-MAPs is the high proportion of missing entries that they contain. Missing entries correspond to pairs of genes for whom interaction strengths could not be measured during the high-throughput process or those that were subsequently filtered due to unreliability. These missing values can reduce the effectiveness of some techniques, *e.g*. introducing instability in clustering [6], and prevent the use of others, *e.g*. matrix factorization techniques such as SVD and PCA. As each epistatic interaction implies a functional relationship between gene pairs, individual epistatic interactions themselves may provide valuable biological insight. Consequently there is an urgent need for an effective imputation technique.

### Related Work

**Figure 2:**
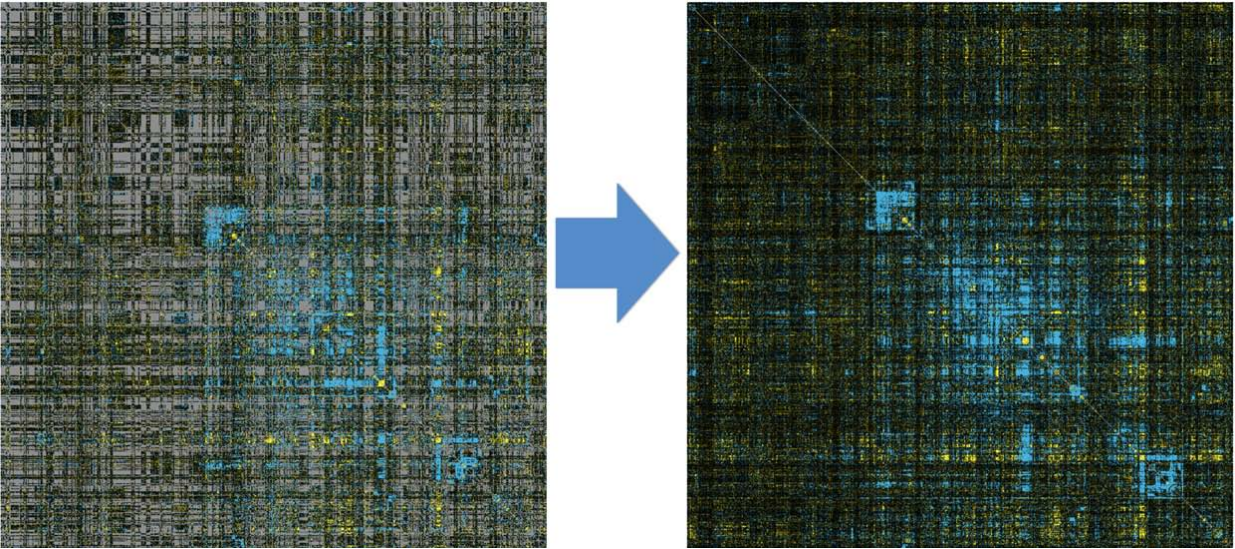
**E-MAP before and after imputation**. A visual representation of a pairwise symmetric E-MAP interaction matrix. On the left-hand side is shown an original E-MAP (Chromosome Biology), where gray points indicate missing values. On the right-hand side is the corresponding complete matrix, with all missing entries replaced by imputed values.

Although the problem of predicting genetic interactions is not new, to our knowledge the problem of imputing quantitative epistasis values in E-MAPs has not previously been evaluated. For E-MAP imputation the goal is to achieve a complete dataset by predicting quantitative scores for all interactions between gene pairs in a given set - including those that display no significant interaction. An illustrative example of an incomplete E-MAP (with missing values) and a corresponding completed E-MAP (with imputed values) is shown in Figure 2.

Järvinen *et al *[7] have applied a matrix approximation technique to a small scale (26 genes) E-MAP-like dataset, and have shown that gene pairs whose growth diverges significantly from the expectation can be identified without the need for measurements of single mutant growth rates. While similar matrix approximation techniques could perhaps be used to address the missing value problem, this was not addressed in their work.

Existing techniques [8-10] focus on predicting binary interactions (synthetic lethality), and work on some operating threshold where only a fraction of all possible interactions are predicted. In other words, they focus on qualitative prediction of the presence or absence of an interaction rather than attempting to quantify the interaction strength. These methods have had some success by mixing heterogeneous biological data [8] or by exploiting the topology of the underlying protein interaction network [9]. More recently Qi *et al *[10] have used graph based methods to predict synthetic lethality, using only the graph of synthetic lethal interactions.

The problem of imputation for E-MAPs more closely resembles that of imputing values in gene expression datasets. The goal in both cases is to construct a complete dataset by imputing quantitative measurements in order to improve the subsequent data analysis. Notably both E-MAPs and gene expression datasets display coherence among genes. For gene expression data this is considered to be indicative of co-regulation, while for E-MAPs it is indicative of co-complex or pathway membership. For this reason E-MAP datasets are typically analyzed using tools developed for gene expression data (*e.g*. the *Cluster *tool [11]) to group together genes with similar interaction profiles as in Figure 1, and to generate heat-maps of the interactions between genes for visual inspection.

The problem of missing value imputation has been well studied for gene expression data. For instance, Troyanskaya and co-workers [12] compared two methods *K*-Nearest Neighbors (KNNImpute) and singular value decomposition (SVD). They recommended KNNImpute as the more robust and accurate method. Since then a number of techniques have been developed, generally falling into two broad categories: local methods, such as nearest neighbor-based techniques, and global methods, generally based on matrix decomposition such as SVD and PCA. In 2008 Brock *et al *[13] provided a comprehensive analysis of different techniques across a number of datasets. Notably, they found that the optimal imputation methods were all competitive with each other, and that the effectiveness of different techniques depended on the "complexity" of the dataset (where the complexity was taken to signify the difficulty with which the data can be reliably transformed to a lower-dimensional subspace). These authors demonstrated that local methods generally performed better on datasets with higher complexity.

### Data Characteristics

Important differences between E-MAP data and gene expression data must be considered:

1. E-MAP datasets are pairwise and symmetric - each missing value represents the interaction between two genes measured under a specific experimental condition, rather than the expression of a given gene in a given sample or at a given time point.

2. E-MAP datasets contain a significantly higher percentage of missing values (up to ≈ 35%), compared with an average of ≈ 5% for gene expression datasets.

3. E-MAP datasets have significantly different dimensionality to gene expression datasets. E-MAPs are symmetric relational datasets (*i.e*. square), typically consisting of between 400 to 800 genes. Gene expression datasets are feature-based (*i.e*. rectangular), frequently containing hundreds or thousands of genes represented across only a small number (*e.g*. 2 to 20) of arrays. This has significant consequences for computational performance when employing matrix factorization techniques.

We observe that there are three types of missing data in E-MAP experiments which may need to be considered separately for the purpose of imputation. Missing values in gene expression datasets are effectively treated as missing at random. This is not the case with E-MAPs where we observe three categories of missing value:

1. *Chromosomal Neighbors: *These consist of gene pairs that are located sufficiently close to one another on a chromosome that recombination events between the two genes are infrequent (within 50 kb for *S.cerevisiae*). Although these pairs are measured in high-throughput experiments, they are removed during a data filtering step because recombination between the relevant genes during the experiment causes an apparent negative interaction that obscures the actual interaction between the pair.

2. *DAmP-DAmP Interactions: *The majority of measured E-MAP interactions arise from complete disruption (deletion) of both genes. In contrast DAmP (Decreased Abundance by mRNA Perturbation) alleles result in unstable mRNAs, and typically are expressed at 5 to 50% of wild type levels [14]. This method is used to disrupt but not completely eliminate the function of essential genes. DAmP - DAmP pairs correspond to combinations of essential genes, which are not generally measured, in part because they grow poorly.

3. *Other Interactions: *This category can be divided into two sub-categories. Firstly, those that correspond to a double mutant measuring the interaction between one essential and one non-essential gene. Secondly, those that correspond to a measurement of the interaction between two non-essential genes. These cases make up the majority of the missing values in an E-MAP and can be considered in the same way for imputation purposes. They are not missing systematically, as is the case with the other categories, and can be treated as missing at random. They occur due to problems in growing the necessary mutants, inconsistencies in the results of multiple experiments, or other problems with the experimental technique.

In general ≈ 100% of the DAmP-DAmP interactions and the chromosomal neighbors are missing from the E-MAP score matrices (see 'Additional file 1 - missing by dataset.pdf'). This means that, although we can impute values for these interactions, we have no effective means of verifying our imputations. Since the third category makes up the majority of the missing values in every published E-MAP, and our predictions for this category can be verified, we focus on this category for the rest of the paper.

## Methods

In this paper we consider four general strategies for imputing missing values in real-valued data - three local methods (nearest neighbor-based) and one global method (BPCA) - and adapt these strategies to work with symmetric data such as E-MAPs.

### Materials

In our evaluations we consider five E-MAPs that have been recently published. These datasets differ in their size, the subset of genes that are studied, and the proportion of missing values that they contain. Four are from the budding yeast *Saccharomyces cerevisiae*, and one is from the fission yeast *Schizosaccharomyces pombe*.

1. **Chromosome Biology: **The largest of the E-MAPs under consideration, this dataset focuses on genes involved in various aspects of chromosome biology, such as DNA replication [3].

2. **RNA Processing: **Focuses on RNA processing pathways [15].

3. **Early Secretory Pathway (ESP): **Focuses on genes whose products are localized to, or have an effect on, the yeast early secretory pathway [14].

4. **Signalling (Kinase): **Focuses on the yeast phosphorylation network, includes the genetic interactions between virtually all kinases and phosphotases [16].

5. **Pombe: **An E-MAP of the fission yeast *Schizosaccharomyces pombe*, emphasizing chromosome function and RNA machinery. This E-MAP was created so that comparisons could be made with an analogous E-MAP in *Saccharomyces cerevisiae *[17].

Table 1 shows the details on the number of alleles, the percentage of missing values, and the total number of measured interactions in each E-MAP.

**Table 1:** Overview of the E-MAPs considered.

*Dataset*	Number of Alleles	Percentage Missing	Measured Interactions
Chromosome Function	754	34.30	187,000
Early Secretory Pathway	424	7.31	83,000
Signalling(Kinase)	483	12.70	102,000
RNA	552	29.54	107,000
Pombe	551	21.75	119,000

Composition of the missing values for the E-MAPs studied, in terms of the percentage from each of the three categories of missing value.

As previously discussed, E-MAPs consist of three distinct categories of missing value. Table 2 shows the composition of the missing values for each of the E-MAPs listed above.

**Table 2:** Composition of the missing values for the E-MAP.

*Dataset*	Neighbors	DAmP-DAmP	Other
Chromosome Biology	2.5	2.7	94.8
RNA Processing	2.9	28.1	69.0
Early Secretory Pathway (ESP)	11.4	24.2	64.4
Signalling (Kinase)	6.4	7.6	86.0
Pombe	4.0	0.3	95.7

Composition of the missing values for the E-MAPs studied, in terms of the percentage from each of the three categories of missing value.

### Method: Filling-in With Zeros

As noted previously, E-MAP interaction datasets are typically normalized so that a data value close to zero indicates the absence of any interaction between a pair of genes. Therefore a simple solution to the problem of missing values is to replace those entries with zeros. While this may appear to be a naïve approach, it has some justification: the expectation is that most genes do not interact, and therefore their interaction score is likely to be close to zero. We also observe that the mean of the non-missing entries in the five E-MAP datasets described previously is approximately zero. This approach serves as a baseline for our experimental evaluations in the next section. Alternative baseline approaches are discussed in the 'Additional file 2 - alternate methods.pdf '

### Method: Symmetric Unweighted *K*-Nearest Neighbors (uKNN)

**Figure 3:**
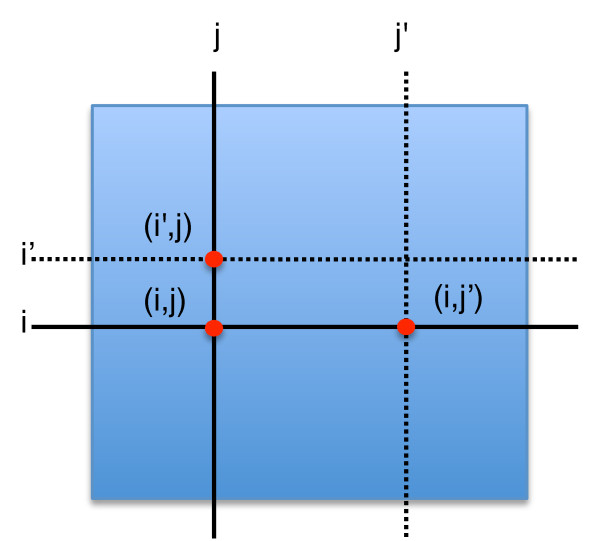
**Symmetric KNN**. Illustration of the symmetric KNN imputation process for parameter *K *= 1. To estimate the missing value (*i*, *j*), the values given by (*i'*, *j*) and (*i*, *j'*) would be combined.

*K*-Nearest Neighbors neighbors (KNN) imputation is a local strategy that uses genes with similar interaction profiles to impute missing values. Standard imputation algorithms based on KNN involve imputing values in feature-based asymmetric datasets. Our proposed approach is designed to handle symmetric data. For each missing interaction (*i*, *j*), we find the *K *nearest neighbor(s) for both gene *i *and gene *j*. We then find the values for the interaction of *i *with *j*'s neighbors, and *j *with *i*'s neighbors. These values are averaged to provide an imputed value for the missing entry (*i*, *j*). An illustration of this approach is shown in Figure 3. For E-MAP data we suggest the use of Pearson's correlation measure to calculate the similarity of gene profiles, as initial experiments indicated that Euclidean distance offered significantly worse performance (data not shown). Note that the effectiveness of this method is heavily dependent on the choice of value for the parameter *K*. Therefore in our experiments we assess the results for a variety of values of *K*.

### Method: Weighted Symmetric Nearest Neighbors (wNN)

Our second proposed approach is similar to the KNN variant described above, but differs in that the contribution of each neighbor to the imputed value is weighted by its similarity to the query gene. Consequently more similar genes make a greater contribution to the imputation. The degree of contribution will be determined by the choice of weighting system. KNNImpute, the KNN imputation approach implemented in [12] for gene expression data, weights genes in direct proportion to their similarity. Troyanskaya *et al *found that this approach was still sensitive to the choice of the parameter k, and initial experiments with E-MAPs confirmed this (see 'Additional file 3 - knnimpute.pdf'). Instead, we employ the following weighting system described in [18], which is similar to a Gaussian kernel function and ensures that closer neighbors are considerably more influential than more distant neighbors. Given a value *r *denoting the Pearson correlation between a gene *i *and its neighbor *i'*, the weight *w*(*i*, *i'*) is calculated as follows:(1)

Note that ϵ is a small value (*e.g*. ϵ = 10^6^) included to avoid a division by zero.

Observe that as the correlation *r *approaches 1, the denominator approaches 0, thereby increasing the weighting dramatically. Thus the weight (and impact) of a neighbor decays dramatically a the correlation drops. As an example, when *r *= 0.9, the associated weight would be *w *≈ 18. While with *r *= 0.5, the resulting weight would only be *w *≈ 0.11. In practice all weights calculated with Eqn. 1 are normalized to sum to one prior to applying the imputation process. The impact of this weighting is that the notion of locality is defined by correlation rather than by the number of neighbors. This overcomes a problem with KNN where poorly correlated neighbors can turn up in the top *K *and have an influence when it is not justified. The weighting strategy has the added advantage that the sharp decay in weight as correlation drops makes wNN significantly less dependent on *K*.

### Method: Symmetric Local Least Squares

Least squares methods have proved effective in imputation for gene expression data [13]. Here we adapt one of the best performing techniques - local least squares (LLS) [19]. This technique involves two steps: the first step is to identify the *K *most similar genes, as in the nearest neighbor techniques, the second is to perform multiple regression on these genes in order to estimate the missing values. The multiple regression represents a target gene as a linear combination of its nearest neighbors as follows:(2)

where *α*_*k *_represents the *k*^*th *^nearest neighbor, and *x*_*k *_is the regression coefficient corresponding to that neighbor. The regression coefficients determine the contribution of each gene to the imputation. This contribution can be negative or positive, and is determined using a least squares formulation (see Kim *et al *[19] for full details). Determination of these coefficients requires an initial estimate for the missing values - in the original implementation these were set to row-averages. In order to adapt this method to work with symmetric data, we perform similar adjustments to those made for KNN. For each missing value (*i*, *j*) an estimate is generated by performing multiple regression on both *i*'s nearest neighbors, and *j*'s nearest neighbors. These two estimates are averaged to produce the final estimate. Similarly, for the purposes of calculating the regression coefficients, the missing value (*i*, *j*) will be initially imputed by averaging the mean interaction score for *i *and *j *across all other genes

### Method: Bayesian Principal Components Analysis (BPCA)

Bayesian Principal Components Analysis is a global imputation approach, which has been shown to be effective for gene expression data [13,20]. The approach involves three steps: principal component regression, Bayesian estimation, and an expectation maximization step. Missing values are initially set to the row mean, and then a probabilistic model for the data and the latent values found within it are iteratively estimated. To make the approach suitable for application to symmetric data, we make a simple intuitive alteration to the algorithm proposed by Oba and colleagues [20]. Specifically we produce a single imputed score for each unique missing pair of genes by averaging the two values, (*i*, *j*) and (*j*, *i*), which are produced by BPCA and may potentially differ in value. A key parameter required by standard PCA approaches is the number of principal axes used for regression. However, BPCA features an automatic relevance determination (ARD) prior, which suppresses the impact of redundant axes. Oba *et al *[20] suggest setting the number of principal axes to *D*-1, where *D *is the number of samples in the dataset, as redundant axes will have lengths of almost zero. This approach is not computationally feasible for E-MAP datasets, due to the much larger dimensionality, so we tried varying number of axes up to a maximum of 300.

In our experiments we used a custom Python implementation of the symmetric uKNN, wNN and LLS imputation approaches available in 'Additional file 4 - emap_imputation.zip' and online at [21]. For the symmetric BPCA approach we used a modified version of the Matlab implementation [22] of the technique proposed by Oba *et al*. [20].

### Assessing the accuracy of quantitative imputations

To assess the effectiveness of imputation techniques for gene expression data, a common approach is to construct a complete matrix from an existing expression dataset by removing those genes which contain missing values. Artificial missing values are then introduced to these complete matrices so that the accuracy of the imputation can be measured. However, this methodology is not directly applicable to E-MAPs for a number of reasons:

1. Each missing interaction would require removal of two genes, rather than a single gene.

2. All DAmP genes would have to be removed, as almost all DAmP - DAmP pairs are missing. This would change the overall nature of the E-MAP significantly, because the inclusion of essential genes is one of the strengths of the technique.

3. The high percentage of missing values makes the methodology impractical. In gene expression experiments typically less than 5% [18] of the values are missing, so genes and arrays can be removed without significantly reducing the size of the dataset. This is not the case for E-MAPs.

Instead we employ an alternative methodology that is more appropriate for E-MAP data. We take an existing incomplete E-MAP matrix, and artificially introduce an additional 1% of missing values. This process can be repeated multiple times so that a large number of imputations are generated, whose accuracy can measured. For our experiments this analysis was carried out 20 times - for a maximum of ≈ 37, 000 interaction scores in the largest dataset and a minimum of ≈ 16, 000 scores in the smallest dataset.

Imputation accuracy can be measured in a number of ways. We consider two measures here in our evaluations. The first is the Pearson correlation between the predicted and actual interactions. The second is the normalized root mean squared error (NRMSE) measure [20] as given by:(3)

where *ij*_*answer *_denotes the set of known values, and *ij*_*guess *_denotes the corresponding set of predicted values. More accurate imputations will result in a higher correlation score, and a lower NRMSE score.

### Assessing the accuracy of strongly alleviating and aggravating interactions

Previous studies have suggested that the accuracy of different imputation techniques is not uniform across all measured values. In particular extreme values can be harder to impute accurately using KNN [23]. In the case of E-MAPs, interactions which have extreme scores are those that are of most interest to biologists, as they indicate strongly alleviating or aggravating interactions between gene pairs.

Using thresholds previously defined in [16] for strongly alleviating (*score >*2.0) and aggravating (*score < -*2.5) interactions, we can partition the data into three distinct interaction classes and assess the performance of our imputation methods as classifiers - *i.e*. in terms of precision and recall. As strong genetic interactions are relatively rare events (less than 10% of all interactions in each dataset considered) we assess classification accuracy over the entire dataset, using 20 fold cross validation, to provide us with as many test points as possible.

Precision and recall are given their standard definition as follows:(4)

In addition we use the *F*_1 _measure as a summary measurement for both precision and recall:(6)

### Assessing the enrichment of imputed interactions for shared annotations

Our ultimate goal is to augment the network of reliable epistatic interactions, so that they may be of use to biological researchers. Therefore we also ask whether the annotated biological properties associated with our imputed gene pairs were similar to those observed for experimentally determined interactions.

It has previously been observed that epistatically interacting gene pairs are more likely to share biological annotations than randomly selected gene pairs [24]. For instance, gene pairs that show strong epistatic interactions are likely to be involved in common biological pathways, and so are likely share Gene Ontology [25] annotations, and will display similar phenotypes. If our imputed epistatic interactions are accurate, we would expect that they would be similarly enriched for shared annotations and phenotypes. To validate our imputations, we considered each class of interaction separately - alleviating, neutral, random - and tested to see if they were more likely to share an annotation than randomly selected pairs from the imputed space. We use two standard resources to form our annotations - Gene Ontology terms and shared phenotypes.

The GO Slim mapping at the Saccharomyces Genome Database (SGD) [26] was used as the source of gene ontology annotations. These are very high-level terms, so annotations which contained more than 1000 genes were filtered out. Phenotype data was also taken from the Saccharomyces Genome Database. Phenotypes associated with more than 175 genes were filtered out, resulting in the removal of terms such as 'inviable', 'viable', and 'haploinsufficient'. Both annotation sets were downloaded on 1^st ^February 2010.

## Results and Discussion

### Choosing Parameters

**Figure 4:**
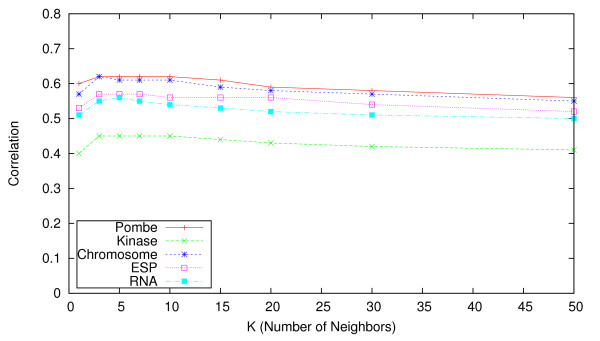
**Effect of K on the accuracy of uKNN**. Impact of choice of value for parameter *K *on imputation accuracy (in terms of correlation) for **KNN **approach.

**Figure 5:**
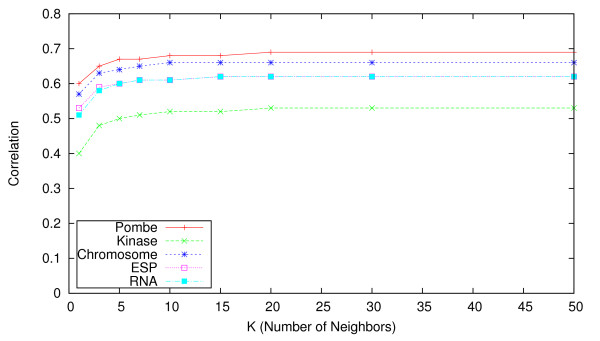
**Effect of K on the accuracy of wNN**. Impact of choice of value for parameter *K *on imputation accuracy (in terms of correlation) for **wNN **approach.

**Figure 6:**
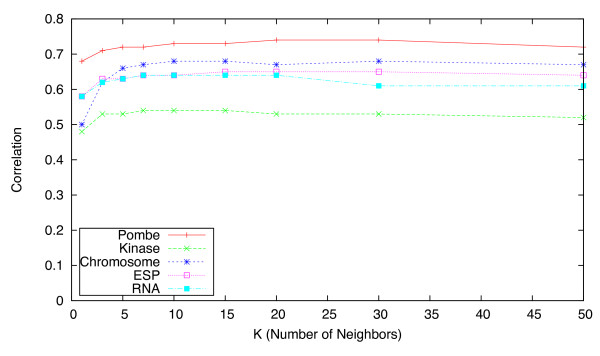
**Effect of K on the accuracy of LLS**. Impact of choice of value for parameter *K *on imputation accuracy (in terms of correlation) for **LLS **approach.

When employing nearest neighbor-based methods, a natural question arises regarding how to choose the number of nearest neighbors *K*, and whether the accuracy of the imputation procedure is sensitive to this choice. In our experiments we considered a range of values for *K *∈ [1, 500]. To illustrate this in Figures 4, 5 and 6 we show the effect of varying *K *up to *K *= 50, for the uKNN, wNN, and LLS approaches respectively.

In the former plot we see that accuracy for unweighted KNN is heavily dependent on a suitable choice for *K*. In contrast, the latter plot shows that, for the weighted KNN variant, the choice of a value for *K *is relatively unimportant for *K >*20 across all five E-MAPs. Our experiments indicated that even with *K >*300 the performance does not degrade. Adding additional neighbors does not have a big impact on computation time, so we suggest that a high value (*e.g. K *≥ 50) could be used as a default when performing imputation on other E-MAP datasets.

LLS displays some sensitivity to K, but is quite stable for 7 *< K <*30. This is unsurprising, as multiple regression contains an implicit weighting scheme - neighbors which explain more of the variance will be given larger regression coefficients, and consequently contribute more to the imputation. Performance starts to degrade for *K >*50 (see 'Additional file 5 - lls large k.pdf'), indicating that local features are more important than global features for imputation in E-MAP datasets. Setting *K *= 20 offers near optimal performance in each dataset, so we suggest its use as a default parameter.

The authors of the original LLS algorithm developed a heuristic to predict a near optimal parameter for *k *-this worked by leaving known values out and attempting to impute them with varying values of *k*. A similar approach could be developed for E-MAPs.

**Figure 7:**
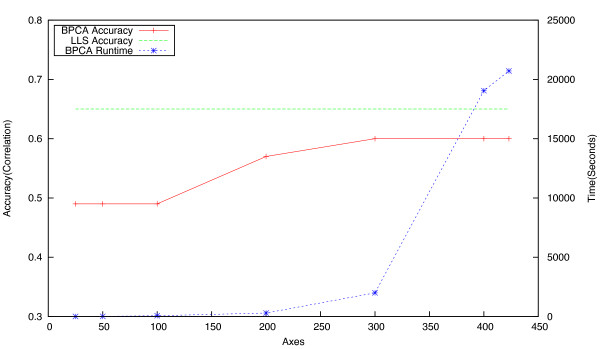
**Effect of the number of axes used on the accuracy and runtime of BPCA (ESP dataset)**. Impact of choice of value for the number of axes on the imputation accuracy (in terms of correlation) and runtime of the **BPCA **approach. Accuracy of LLS is shown for comparison, with *K *= 20. Running time is averaged across twenty runs. Note that these experiments were run on a 20 core machine with 128GB RAM, using all cores at 100%. Computation time on a standard desktop machine would therefore take substantially longer.

**Figure 8:**
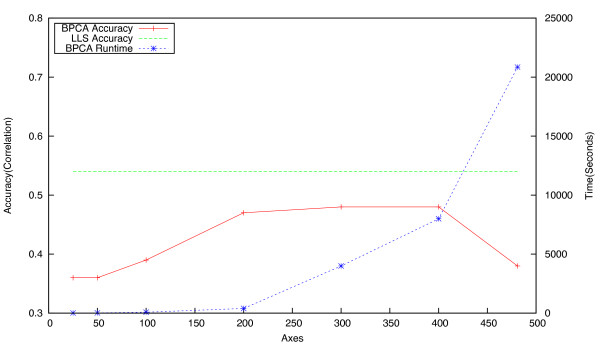
**Effect of the number of axes used on the accuracy and runtime of BPCA (Signalling dataset)**. Impact of choice of value for the number of axes on the imputation accuracy (in terms of correlation) and runtime of the **BPCA **approach. Accuracy of LLS is shown for comparison, with *K *= 20. Running time is averaged across twenty runs. Note that these experiments were run on a 20 core machine with 128GB RAM, using all cores at 100%. Computation time on a standard desktop machine would therefore take substantially longer.

For BPCA, it is not only the accuracy of the imputation procedure which needs to be taken into account, but also its computational tractability. To investigate this issue, BPCA imputation was attempted on the two smallest datasets (ESP and Signalling), using a range of axes from 25 to *D *- 1. Accuracy and running time figures for these experiments are given in Figures 7 and 8. Note that beyond 300 axes, the time increases dramatically, while accuracy does not increase significantly. When applied to the largest dataset (Chromosome Biology) with the number of axes set to *D *- 1, BPCA took approximately one week to converge on a solution, and more frequently did not converge at all. This is unsurprising given the large fraction of missing values in this dataset, and the high number of principal axes computed, both of which have a significant impact on the algorithm's computational performance. As a consequence of this infrequent convergence and the time taken to run the procedure, experiments on the Pombe, RNA and Chromosome datasets were carried out with the number of axes set to a maximum of 300.

### Performance across different datasets

Tables 3 and 4 respectively show the correlation and NRMSE accuracy scores for all imputation approaches, along with the baseline method of filling-in with zeros. Of the range of methods evaluated in our experiments, LLS demonstrated the best accuracy figures for all datasets, with wNN a close second. A two-tailed paired t-test of the errors for each method indicated that there was a statistically significant difference between LLS and wNN on the ESP, Chromosome and Pombe datasets(*p *< 10^-8 ^in all cases), while for the RNA and Signalling datasets there was no significant difference.

**Table 3:** Accuracy, as measured by correlation, across five E-MAPs.

*Approach*	Pombe	Kinase	Chromosome	ESP	RNA
Filling with zeros	0.00	0.00	0.00	0.00	0.00
uKNN (K = 5)	0.64	0.45	0.61	0.57	0.56
BPCA(K = 300)	0.68	0.48	0.53	0.61	0.58
wNN (K = 50)	0.71	0.53	0.66	0.62	0.62
LLS (K = 20)	**0.74**	**0.54**	**0.68**	**0.65**	**0.64**

Results of further experiments, comparing all approaches on five E-MAPs. Accuracy scores are given in terms of predicted/actual value correlation.

**Table 4:** Accuracy, as measured by NRMSE, across five E-MAPs.

*Approach*	Pombe	Kinase	Chromosome	ESP	RNA
Filling with zeros	1.01	1.00	1.01	1.00	1.00
uKNN (K = 5)	0.78	0.90	0.79	0.83	0.83
BPCA(K = 300)	0.74	0.89	0.85	0.80	0.82
wNN (K = 50)	0.71	0.85	0.75	0.79	0.78
LLS (K = 20)	**0.68**	**0.85**	**0.73**	**0.76**	**0.77**

Results of further experiments, comparing all approaches on five E-MAPs. Accuracy scores are given in terms of normalized root mean squared error (NRMSE).

While BPCA is an improvement on KNN, we observe that it fails to match the performance of either wNN or LLS - even on the ESP and Signalling datasets where parameters were evaluated across a broad spectrum. A two-tailed paired t-test of the errors for each method confirmed that there was a statistically significant difference in performance on all datasets between both wNN and BPCA, and LLS and BPCA. As BPCA does not offer any improvement in accuracy, and because it is impractical to use on larger datasets, we do not recommend it for E-MAP imputation. In all subsequent analysis we focus on the two most competitive imputation procedures - wNN and LLS.

Both of these local procedures demonstrated good performance across the majority of the datasets, albeit with significantly poorer results when applied to the Signalling E-MAP. This perhaps arises due to the nature of this particular dataset. Generally E-MAPs focus on genes involved in a general biological process, leading to coherence in the datasets (genes involved in the same pathway or complex tend to display similar interaction profiles). In contrast the Signalling E-MAP contains kinases and phosphatases from a wide variety of locations and processes in the cell, and therefore does not contain as many coherent complexes or pathways. Indeed, in the associated work [16], the primary analysis was not performed with clustered heat-maps, but rather using topological features of the network combined with mapping of the genetic interactions onto known pathways. One future application of our approach might include introducing such additional information to improve the imputation.

There is no obvious connection between the percentage of missing values present in a dataset and the accuracy of any of the imputation approaches - indeed performance is better on the largest (Chromosome) dataset than it is on the smallest (ESP) dataset. One explanation for this is that, even with a larger percentage of missing values, the Chromosome dataset contains more information overall. A second explanation is that in the larger datasets there are a larger number of neighbors to choose from for the purpose of imputation.

Additional experiments also indicate that there is no obvious connection between the number of missing interactions for an individual gene and the accuracy of imputation on its missing values. For example, in the RNA dataset, genes with 50-60% missing values are imputed with higher accuracy than those with 10 - 20% missing values. See 'Additional file 6 - missing by percentage.xls' for full details. This is perhaps surprising, but in E-MAP datasets even genes with ≈ 60% missing values have several hundred measured values which can be used to identify nearest neighbors. This is in contrast with gene expression data, where the number of measurements can be lower than 12 per gene. This may have important consequences for optimizing the design of pairwise genetic interaction studies. Previous work by Casey *et al *[27] showed that using by combining an iterative experimental approach with information theory approaches to identify the most informative experiments, successful clustering of interaction data could be achieved using less than 50% of the measurements in a complete dataset. It would be interesting to see a similar approach based on optimal imputation of strong interactions.

### Strongly alleviating and aggravating interactions are imputed with high precision

Although the stated purpose of this work is not to develop classifiers for alleviating or aggravating interactions, the classification results are still of some interest. Figures for the classification accuracy of the three distinct classes of interaction (Alleviating, Neutral, Aggravating) are given in Table 5. These figures were generated with our suggested default parameters - *K *= 50 and *K *= 20 for wNN and LLS respectively. The precision and recall figures for aggravating interactions shown are competitive with recently reported findings in [10] for the prediction of synthetic lethality. However, the results for alleviating interactions are significantly poorer. This is surprising, but to date there have been no methods developed for the prediction of alleviating interactions with which to make a comparison. There are a number of possible explanations for the poorer recall - there are fewer measured alleviating interactions in each dataset, and they generally have a smaller magnitude. In addition, the biological factors which result in alleviating interactions have not been the subject of as many systematic studies as those of aggravating interactions. We suggest that this is an area in which significant further work can be done - both in terms of improving predictive accuracy, and also gaining an understanding of the causes of alleviating interactions.

**Table 5:** Classification accuracy comparisons (in terms of precision, recall and *F*_1 _scores) for the strongly aggravating and alleviating classes of interactions found in E-MAPs.

*Dataset*	*Method*	Alleviating			Aggravating		
		
		Precision	Recall	*F*_1_	Precision	Recall	*F*_1_
Chromosome	*wNN*	**0.66**	**0.14**	**0.23**	0.71	**0.40**	**0.51**
	*LLS*	0.65	0.07	0.13	**0.74**	0.38	0.50

RNA	*wNN*	0.69	**0.14**	**0.23**	**0.72**	**0.39**	**0.51**
	*LLS*	**0.75**	0.11	0.19	**0.72**	0.35	0.47

Pombe	*wNN*	0.64	**0.17**	**0.27**	**0.70**	0.49	**0.58**
	*LLS*	**0.74**	0.09	0.16	0.69	**0.50**	**0.58**

Signalling	*wNN*	**0.71**	**0.06**	**0.11**	**0.65**	0.27	0.38
	*LLS*	0.50	0.01	0.02	**0.65**	**0.32**	**0.43**

ESP	*wNN*	**0.78**	**0.14**	**0.24**	0.64	**0.42**	0.51
	*LLS*	0.67	0.09	0.16	**0.66**	**0.42**	**0.52**

The highest value for each dataset is highlighted in bold. While the neutral class has been left out for the sake of clarity, note that in all cases recall was ≈ 0.99 and precision was > 0.96.

While precision scores are competitive for both LLS and wNN, we note that wNN offers better recall in most cases. One possible explanation is that each method selects the neighbors in a slightly different fashion - for a missing value (*i*, *j*), wNN selects only *i*'s K nearest neighbors that have a measured interaction with *j*, while LLS selects K neighbors based solely on correlation. This is done for reasons of efficiency in LLS - regression coefficients are calculated for each gene with missing values, rather than for each missing value. Some of *i*'s K nearest neighbors may have a missing value for the interaction with *j *- in LLS these are filled in with gene mean values and used for the imputation, while for wNN these neighbors will be skipped and the next most similar neighbors selected. The fact that LLS sometimes uses values imputed using means will have a greater impact when dealing with extreme values, as the gene mean values represent a poor estimation for them.

As discussed in the methods section, these results are generated by artificially introducing missing values to the E-MAPs. However, consistent with the higher recall reported here, when imputation is applied to the actual missing values in E-MAPs, wNN predicts a larger number of strongly alleviating and aggravating interactions. For example - within the Chromosome Biology E-MAP wNN predicts 1450 aggravating and 190 alleviating interactions, while LLS predicts only 988 and 97 for the same categories.

### Imputed epistatic interactions are enriched for shared annotations

Our ultimate goal is to augment the network of reliable epistatic interactions, so that they may be of use to biological researchers. Therefore we next asked whether the annotated biological properties associated with our imputed gene pairs were similar to those observed for experimentally determined interactions.

**Figure 9:**
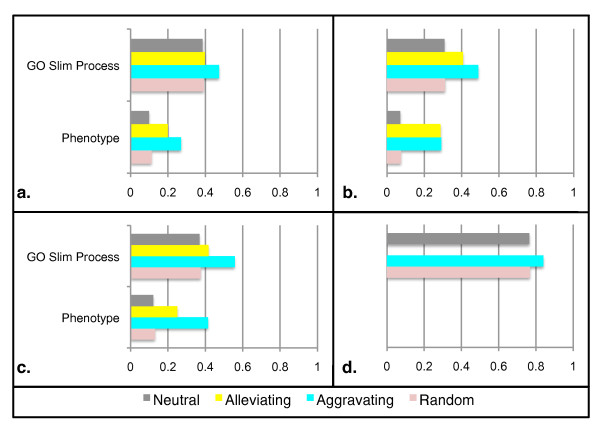
**Fraction of each class of interaction which share an annotation (Chromosome E-MAP using wNN)**. **a**. Measured interactions, **b**. All imputed interactions, **c**. Imputed Chromosomal Neighbors, **d**. Imputed DAmP-DAmP pairs.

Figure 9 shows the result of this enrichment analysis on one dataset (Chromosome Biology) - as with measured interactions(a), both aggravating and alleviating imputed gene pairs are more likely to share an annotation than randomly selected gene pairs (b). For all cases this enrichment was statistically significant (*p *< 0.01 using Fisher's exact test). Furthermore, we tested the imputed interactions between "chromosomal neighbors"(c) and "DAmP-DAmP" pairs(d). For the "chromosomal neighbor" class we found that both alleviating and aggravating interactions were enriched for shared annotations, but only the aggravating interactions were enriched at a statistically significant level. Since only one of the DAmP-DAmP pairs was predicted to have an alleviating interaction, alleviating interactions are not included in chart (d). The aggravating interactions were enriched, but not at a statistically significant level. We note that even randomly selected DAmP-DAmP pairs are significantly more likely to share an annotation. We surmise this is because essential genes are better annotated. Phenotype data was excluded from the DAmP-DAmP analysis, as the annotations largely come from knock out studies, where the phenotype for DAmP genes would be 'inviable'. These results were generated using the wNN imputation approach 'Additional file 7 - lls enrichment.pdf' shows results for the same analysis using LLS imputations, which were similarly enriched, although at a slightly less significant level. In addition - 'Additional file 8 - esp enrichment.pdf' shows the similar trends when the same analyis is applied to the ESP dataset. Overall, both internal (leave-one-out analysis) and external (comparison with annotated biological features) validation support the view that our imputation procedures generate reliable predictions for novel epistatic relationships of both positive and negative polarity.

### Impact of imputations on downstream analysis

One of our motivations for imputation in E-MAPs is to improve downstream analysis. A widely used downstream analysis technique applied to E-MAP data is average-linkage hierarchical clustering, using the *Cluster *[11] tool. This groups together genes that have similar interaction profiles, and is used to identify genes whose products are part of the same physical complex or pathway [3,14]. In order to assess the impact of our imputation on clustering and on downstream biological analysis, we compared clusterings on the ESP and RNA datasets before and after imputation using the wNN approach. We used a hypergeometric test to identify clusters that had a statistically significant overlap with known protein complexes. Each node of the tree was compared with each protein complex, and p-value assigned to this overlap. Multiple comparisons were corrected for using the Bonferonni correction, and our significance threshold was set to *p *< 0.05. The list of known complexes was taken from an up to date manually curated list [28], which contains 408 complexes with reliable evidence from small scale experiments. In the RNA dataset we identified the same twelve complexes before and after imputation. However five of these (COMPASS, Prp19-associated complex, SAGA, U1 snRNP complex, commitment complex) are identified with increased precision at the same, or higher, level of recall. See 'Additional file 9 - significant clusters.xls' for details of the complexes found. In the ESP dataset we identified six complexes with statistical significance prior to imputation, while after imputation we found clusters enriched for the same six complexes, together with an additional one - the ubiquitin ligase ERAD-L complex(a protein complex with ubiquitin ligase activity involved in degradation of misfolded proteins in the endoplasmic reticulum), three of whose members formed a single cluster. These examples demonstrate that the inclusion of imputed values can improve precision and recall characteristics of a clustering analysis of annotated protein complexes, thereby facilitating downstream biological analysis.

### Applicability to other data

The methods discussed here are intended for use with large scale quantitative genetic interaction data. To date, alternatives to E-MAPs have generally created datasets which are of large scale but binary in nature [29] or small scale but quantitative [30]. However an increasing amount of large scale quantitative interaction data is anticipated, for instance from the forthcoming database of quantitative interactions in yeast [31]. Our results show that local E-MAP imputation methods work effectively in data obtained in two different species, *Schizosaccharomyces pombe *and *Saccharomyces cerevisiae*. Although the experimental technique used for both organisms uses the same basic experimental design and format, they are widely divergent in terms of genome structure and evolution(≈ 400 million years). This result is reassuring because it indicates that techniques developed for application to one organism may be effective for analogous techniques developed in another. One such technique is GIANT-coli [32], which measures quantitative genetic interactions in the bacteria *Escherichia coli*. To date the largest available dataset resulting from this method is a 12 × 12 matrix, however larger datasets are expected. Screening methods for synthetic genetic interactions have also been developed for the worm *Caenorhabditis elegans *[33].

### Further Work

There are a number of areas not addressed in this paper which merit further work. One issue is the accuracy of predictions for the two categories of missing data not addressed by this paper: DAmP - DAmP pairs and chromosomal neighbors. We have shown that strongly interacting gene pairs from these categories are enriched for shared annotations typical of experimentally measured genetic interactions, but we have no data on which to assess their quantitative accuracy. Recent improvements in the experimental tools available to study essential genes [34] should facilitate the measurement of a larger number of pairwise interactions between essential genes, and thus provide a means for assessing the accuracy of imputation on DAmP-DAmP pairs. Smaller scale experiments could also be used to measure the effectiveness of imputation on chromosomal neighbors.

Another avenue for future work would be to examine the degree to which imputation improves the effectiveness of subsequent data analysis procedures when applied to E-MAPs. We have shown that imputation can improve the use of hierarchical clustering to identify known protein complexes, but there are many additional downstream analyses which could be assessed. More interesting, perhaps, will be the analysis of E-MAP data using previously inapplicable methods - such as PCA.

Due to the high number of missing values in E-MAPs, the imputation generates thousands of predictions for novel interactions. It may prove useful to investigate whether any of the imputed aggravating or alleviating interactions are biologically interesting in their own right.

Finally, it may be possible that proposed imputation approaches could be improved by incorporating external sources of information, such as topological features from protein-protein interaction data, gene co-expression data, and subcellular localization.

## Conclusions

We have introduced the problem of missing value imputation for Epistatic MAPs, and provided three categories for the missing values that they contain. We have shown that local imputation strategies are more accurate and much more computationally tractable than global PCA-based strategies. We have proposed three local imputation approaches based on the use of nearest neighbor information. Evaluations performed on a comprehensive set of E-MAPs from two yeast species suggest that in terms of absolute accuracy the local least squares imputation strategy is marginally better than the weighted nearest neighbor strategy with both outperforming the unweighted nearest neighbor approach. However, the weighted nearest neighbor approach is generally better at recalling strongly interacting epistatic gene pairs, suggesting that it may be more useful for those interested in analysis of individual interactions. For these reasons we suggest that both the local least squares and weighted nearest neighbor imputation strategies should be considered for the further analysis of Epistatic MAPs and we have made an implementation of both methods available online. We have also suggested a number of follow-up research topics which should be facilitated by these implementations.

## Authors' contributions

GC identified the problem, suggested gene expression as a starting point. DG, PC and CR proposed the imputation approaches and designed the experimental setup. CR wrote the nearest neighbor implementations and performed the experimental evaluations. All authors read and approved the final manuscript.

## Supplementary Material

Additional file 1 A table in pdf format showing the percentage of each type of data missing in the five datasets. Click here for file

Additional file 2 A table in pdf format, containing accuracy figures for two alternative simple imputation methods - 'Gene Means' and 'Medians'. Click here for file

Additional file 3 **An image in pdf format, showing the accuracy of KNNImpute with respect to choice of *K*.** This was generated using a symmetric implementation of the KNNImpute algorithm described in Troyanskaya *et al*. Neighbors are weighted in direct proportion to their similarity to the query gene. Similarity is measured using correlation. Unlike the weighting scheme we use for our wNN approach, KNNImpute is still very sensitive to the choice of K. Click here for file

Additional file 4 **A zip file containing Python code implementing the nearest neighbor algorithms described in this article, instructions for its use, and a sample input file.** This file is made available in order to ensure that the code is available as long as the journal article. However the authors request that those wishing to use the code visit [21], where any updates to the code will be made available. Click here for file

Additional file 5 An image in pdf format, showing the accuracy of LLS for higher values of K. As K is increased past 50, performance starts to degrade significantly, indicating the importance of local features. Click here for file

Additional file 6 **A table in .xls format, showing the accuracy of imputation on genes with varying percentages of missing values. **Interactions are sorted into bins based on the percentage of missing values in their corresponding genes. An interaction between a pair of genes with 14% and 55% missing values would be counted in both the '10 - 20' and '50 - 60' bins. NRMSE and correlation are then calculated for each bin. These figures are calculated for every interaction in the RNA and ESP dataset - using *K *= 50 and *K *= 20 for the wNN and LLS methods respectively. Click here for file

Additional file 7 **An image in pdf format, showing the fraction of each class of interaction which share an annotation.** Generated on the Chromosome E-MAP, using LLS imputation. Labels are as in Figure 9. Click here for file

Additional file 8 **An image in pdf format, showing the fraction of each class of interaction which share an annotation.** Generated on the ESP E-MAP, using wNN imputation. Labels are as in Figure 9. Click here for file

Additional file 9 **A table in .xls format, showing protein complexes identifed using hierarchical clustering before and after imputation.** Precision, recall and a p-value are given for each cluster which has a statistically significant overlap with a known protein complex. Values which differ before and after imputation are in bold. Click here for file
