# Supplementary material for: Missing value imputation for epistatic MAPs
Source: BMC Bioinformatics. 2010 Apr 20;11:197. doi: 10.1186/1471-2105-11-197 (PMC2873538; doi:10.1186/1471-2105-11-197)
Supplement: Additional file 1 — A table in pdf format showing the percentage of each type of data missing in the five datasets. [file 1471-2105-11-197-S1.PDF]

Percentage of each type of data missing in the 5 datasets

	**Neighbors**	**DAmP-DAmP**	**Other**
Chromosome	99.31	99.10	33.12
RNA	100	95.17	22.54
ESP	100	100	4.83
Signalling	99.79	100	11.12
Pombe	100	100	21.01

Note that the percentage of missing DAmP – DAmP pairs and chromosomal neighbors is near 100% in each dataset. It is unclear why a small percentage of values are present in the RNA, Chromosome & Signalling datasets
