# Supplementary material for: Missing value imputation for epistatic MAPs
Source: BMC Bioinformatics. 2010 Apr 20;11:197. doi: 10.1186/1471-2105-11-197 (PMC2873538; doi:10.1186/1471-2105-11-197)
Supplement: Additional file 2 — A table in pdf format, containing accuracy figures for two alternative simple imputation methods - 'Gene Means' and 'Medians'. [file 1471-2105-11-197-S2.PDF]

Performance of alternative simple imputation methods, as measured by correlation

	**Pombe**	**Signalling**	**Chromosome**	**ESP**	**RNA**
Zeros	0.00	0.00	0.00	0.00	0.00
Medians	**0.34**	0.19	0.25	**0.36**	0.19
Means	0.33	**0.24**	**0.27**	0.18	**0.26**

Performance of alternative simple imputation methods, as measured by NRMSE

	**Pombe**	**Signalling**	**Chromosome**	**ESP**	**RNA**
Zeros	1.01	1.00	1.01	1.00	1.00
Medians	0.97	0.99	0.99	**0.94**	0.99
Means	**0.96**	**0.98**	**0.97**	0.99	**0.97**

Method: Gene Means

This is a naive global technique and represents a simple improvement on filling in with dataset means. For each missing interaction (i,j) we calculate an estimate by averaging the mean interaction score for i and j across all other genes. This is used as an initial estimate for LLS.

Method: Medians

It has been previously observed that there is a relationship between the measured interaction of two genes, and the correlation between their interaction profiles. See Schuldiner *et al*[14] for full details.

Collins *et al* [3] suggested that this could be used as a way to estimate missing genetic interactions by finding the median interaction score for interactions between gene pairs with similar correlations between their interaction profiles.

We implemented this approach as follows : for the missing value (i,j), we calculate the correlation (*r*) between the interaction profiles of i and j. We then identify gene pairs with a similar interaction profile correlation, and find the median interaction score between them. A sliding window of size 0.1 was used to implement this – so for a correlation of 0.65, gene pairs which had a correlation between 0.6 and 0.7 would be used to calculate the median.
