# Supplementary figures and images for: Missing value imputation for epistatic MAPs
Source: BMC Bioinformatics. 2010 Apr 20;11:197. doi: 10.1186/1471-2105-11-197 (PMC2873538; doi:10.1186/1471-2105-11-197)

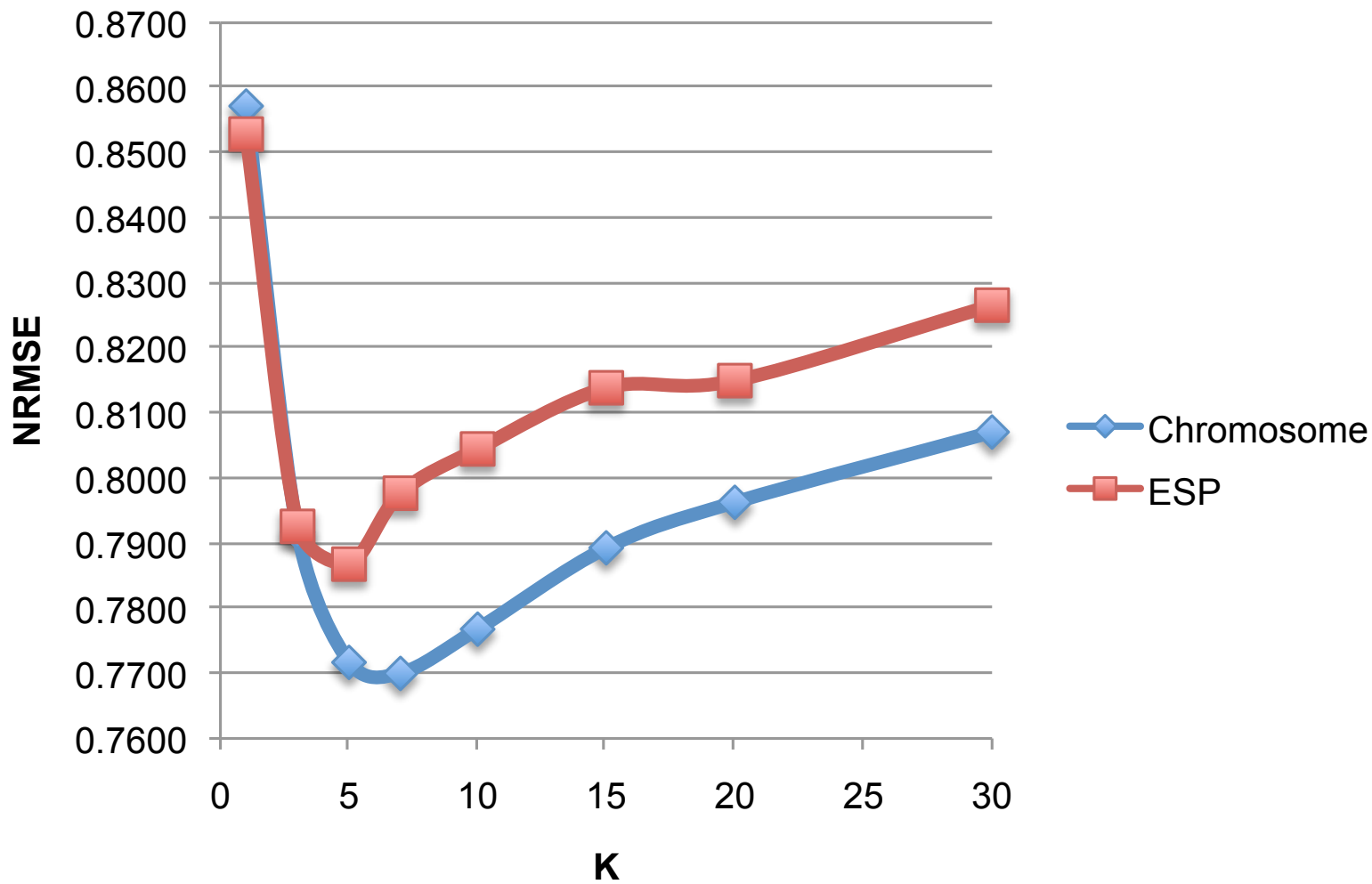

Supplement: Additional file 3 — An image in pdf format, showing the accuracy of KNNImpute with respect to choice of K. This was generated using a symmetric implementation of the KNNImpute algorithm described in Troyanskaya et al. Neighbors are weighted in direct proportion to their similarity to the query gene. Similarity is measured using correlation. Unlike the weighting scheme we use for our wNN approach, KNNImpute is still very sensitive to the choice of K. [file 1471-2105-11-197-S3.PDF]

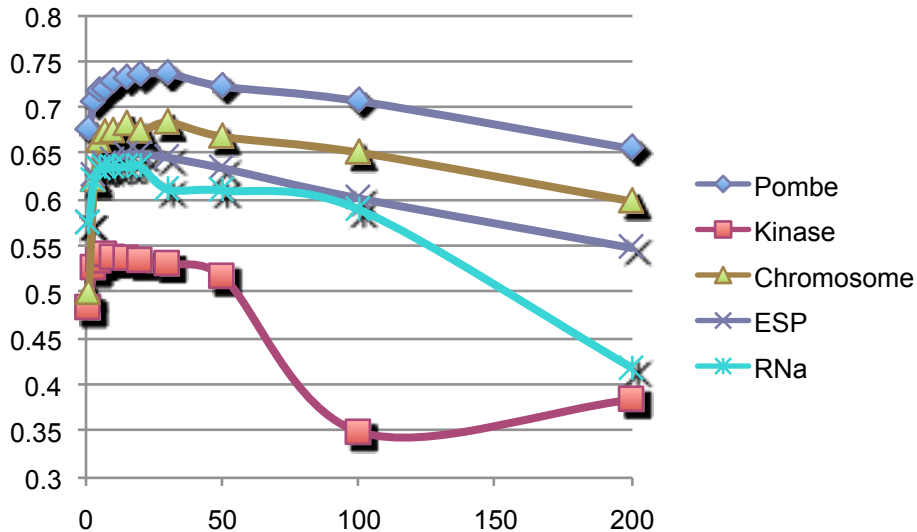

Supplement: Additional file 5 — An image in pdf format, showing the accuracy of LLS for higher values of K. As K is increased past 50, performance starts to degrade significantly, indicating the importance of local features. [file 1471-2105-11-197-S5.PDF]

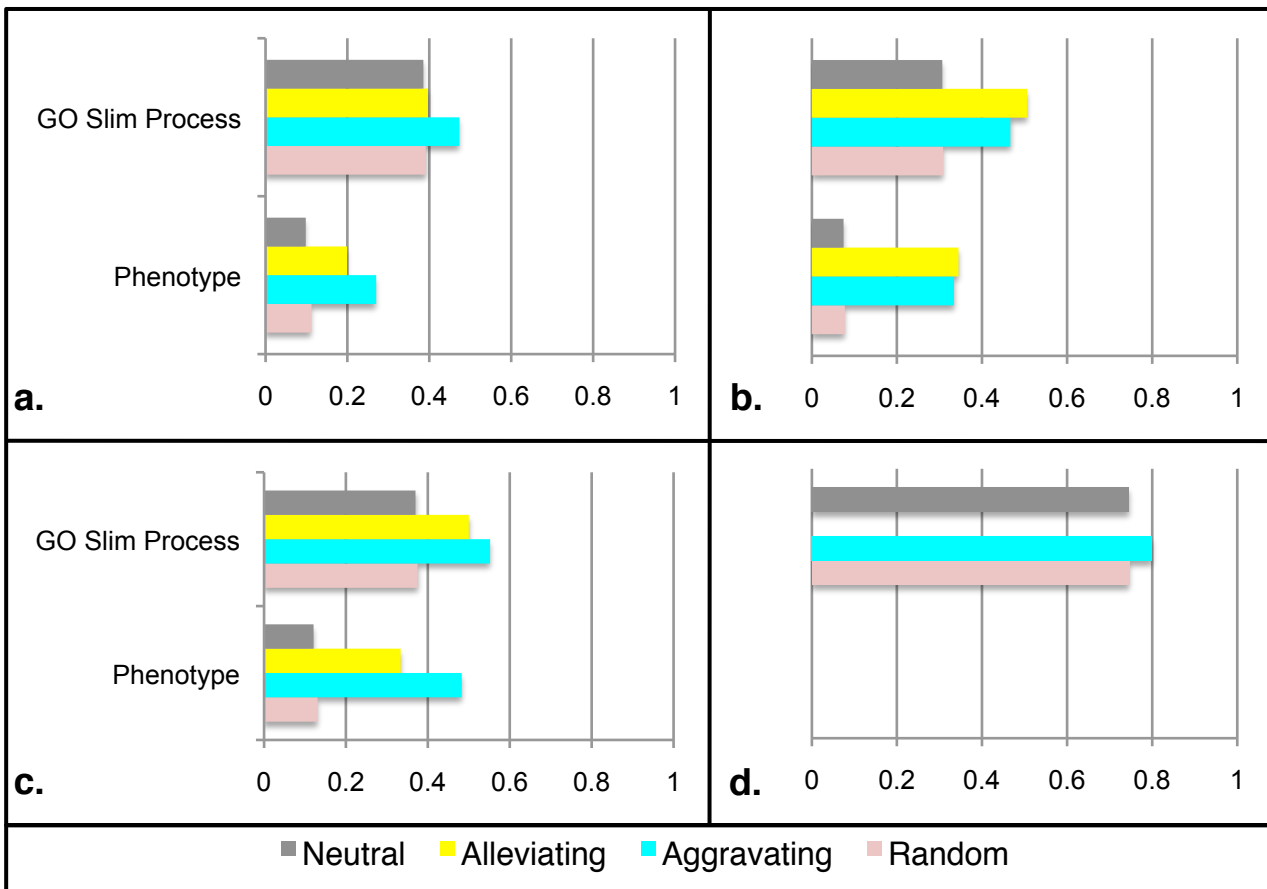

Supplement: Additional file 7 — An image in pdf format, showing the fraction of each class of interaction which share an annotation. Generated on the Chromosome E-MAP, using LLS imputation. Labels are as in Figure 9. [file 1471-2105-11-197-S7.PDF]

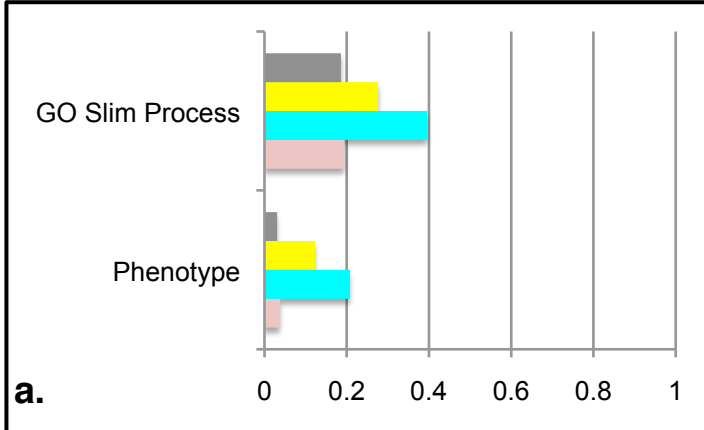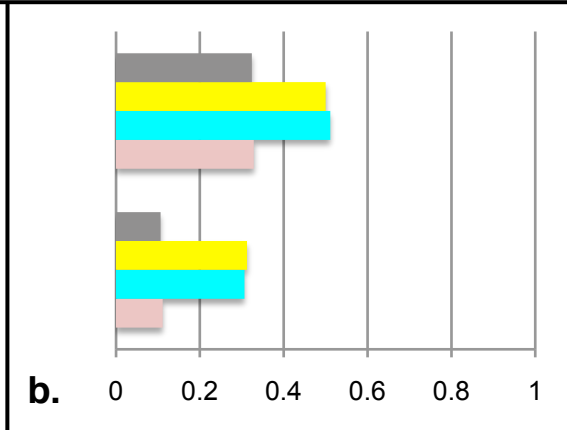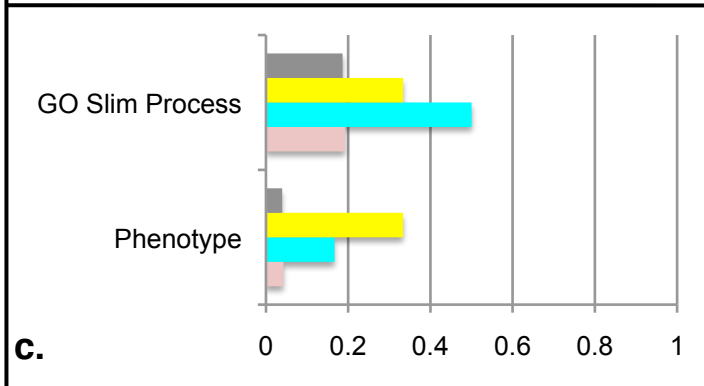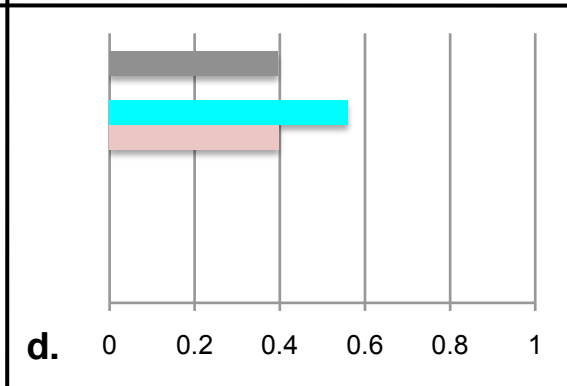

■ Neutral ■ Alleviating ■ Aggravating ■ Random

Supplement: Additional file 8 — An image in pdf format, showing the fraction of each class of interaction which share an annotation. Generated on the ESP E-MAP, using wNN imputation. Labels are as in Figure 9. [file 1471-2105-11-197-S8.PDF]
